# Diminishing returns of task-oriented interaction in digitally-mediated dynamic teams: evidence from amateur sports organizing

**DOI:** 10.3389/fpsyg.2025.1548846

**Published:** 2025-08-18

**Authors:** Jing Yang, Tang Yao, Jun Wang

**Affiliations:** ^1^School of Mathematics, Chengdu Normal University, Chengdu, China; ^2^School of Economics and Management, Beihang University, Beijing, China

**Keywords:** task-oriented interaction, diminishing returns, team identification, team efficiency, dynamic teams, amateur sports organizing

## Abstract

Although extant research has emphasized task-oriented processes in teams, its focus on dynamic teams in digital environments remains limited, particularly regarding non-linear effects. Integrating attention capacity theory and activation theory, this study proposes a curvilinear (inverted-U) relationship between task-oriented interaction and team organizing efficiency in digitally-mediated teams. Analyzing 455 spontaneous sports teams from an open-boundary organizational platform revealed support for the proposed curvilinear relationship, with team identification moderating the effect. Specifically, when team identification was low (vs. high), the inverted-U relationship was more salient. With high (vs. low) team identification, teams exhibited relatively higher levels of team organizing efficiency, regardless of task-oriented interaction. These findings establish diminishing returns of task-oriented interaction as a fundamental boundary condition for digitally-mediated organizing efficacy, advancing theory on dynamic team coordination and offering pragmatic guidelines for managing technology-mediated sports collaboration.

## 1 Introduction

Due to the importance attached to teams in the organizational environment, many researchers have conducted extensive research to identify the key factors that make for successful team outcomes (Humphrey and Aime, 2014). Extant studies have provided valuable insights into the key role of task-oriented processes in stable teams (Anderson and Potočnik, 2014; Braun et al., 2013; Rosing et al., 2011; Van Knippenberg and Sitkin, 2013). However, emerging digital platforms facilitate new forms of dynamic teams—spontaneously formed groups coordinating through technology-mediated interactions (e.g., amateur sports activity platforms; Fenton et al., 2021; Felix et al., 2022)—where classic team models face theoretical challenges (Mortensen and Haas, 2018). In these contexts, the notion of teams as unchanging entities has been challenged in organizational practice, with teams often proving to be evolving and boundary-blurring entities (Li et al., 2018; Mortensen and Haas, 2018), yet research on task-oriented processes remains scarce and methodologically constrained. As new individuals join or existing ones leave, team composition and collaboration strategies can undergo significant changes (Dibble and Gibson, 2018; Summers et al., 2012). Meanwhile, the way of cooperation and coordination has changed as teams rely more on information and collaboration technologies (Larson and DeChurch, 2020; Barley et al., 2017). Shared service and information centers have been used to achieve overall performance improvement in some organizations that have successfully decentralized (Delice et al., 2019). Thus, it is crucial for researchers to understand how teams can develop strategies to effectively adapt to a changing context (Kozlowski and Ilgen, 2006).

Recognizing the dynamic nature of the organizational context raises questions about whether the existing research on stable teams is applicable to dynamically changing teams. Some studies imply that open boundary drives the change in the way a team evolves and performs (Humphrey and Aime, 2014; Marks et al., 2001). However, little is known about the role of task-oriented processes in dynamic contexts. Furthermore, most previous studies relied on weak methodological approaches such as self-report questionnaires and interviews, which can result in unfair comparisons and ecological validity problems that hinder the identification of real causal effects (Hughes et al., 2018; Hauser et al., 2017). Especially in a dynamic context, because of the limitation of the ability to observe all aspects of the measured constructs, participants’ reports may risk the development of inaccurate conclusions. To address this empirically, we conducted a natural field study of 455 spontaneously formed football teams on DaZhi—a digital platform enabling real-time coordination of sports activities. This context epitomizes digitally-mediated organizing in boundary-open teams, where task-oriented interaction focuses on resource broadcasting for event execution rather than deep co-creation. This context embodies three key attributes essential for probing dynamic organizing: (1) theoretically, amateur football teams inherently exhibit fluid membership and spatial dispersion (Dibble and Gibson, 2018), while preserving core team properties like goal interdependence and role differentiation (Kozlowski and Ilgen, 2006)—making them ideal exemplars of boundary-open systems. (2) Methodologically, DaZhi enables unobtrusive tracking of resource-broadcasting behaviors (e.g., activity posting, participant recruitment), generating ecologically valid data absent in self-reports. (3) Empirically, sports teams are established microcosms for organizational research, with football providing high-frequency, observable organizing cycles (Myers et al., 2004).

Our study primarily contributes to task-oriented processes and team efficiency research in three ways. First, despite their existence in and value to boundary-clear teams (Peñarroja et al., 2013), the current is one of the first to explore dynamic and boundary-open teams in digitally-mediated amateur sports organizing contexts. Second, we investigate the curvilinear impact of task-oriented interaction on team organizing efficiency while most previous empirical studies have focused on the linear relationship between them (West and Anderson, 1996; Eisenbeiss et al., 2008). Only one study suggested a curvilinear impact on overall job performance related to a task-oriented process (Zhang and Bartol, 2010), but it focused on creative process engagement and was also based on stable teams. Third, our study integrates research on task-oriented processes and team efficiency to propose a moderated model that links task-oriented interaction and team identification to team organizing efficiency. Despite the importance of team identification in team research (Hogg and Abrams, 1988; Hogg and Terry, 2000), team identification has received little attention in task-oriented process literature.

The remainder is organized as follows. The next section outlines our conceptual framework. The third section develops hypotheses that link task-oriented interaction and team identification to team organizing efficiency. The fourth section discusses our methodology and the fifth section presents the results and robustness testing. The sixth and final sections provide general discussions and conclusions.

## 2 Conceptual framework

Traditionally, team research commonly presumes that team processes, such as team identification (Bezrukova et al., 2009; Van Dick et al., 2004) and task-oriented processes (Anderson and West, 1998; Peltokorpi and Hasu, 2016), are key determinants of many focused issues and outcomes (Postmes et al., 2001; Schneider et al., 2017). To understand the role of information exchange pertaining to performing tasks in digitally-mediated dynamic teams, we focus on task-oriented interaction because of its importance and emphasis on goal achievement (West, 2002; Shalley, 2002). Previous related research has used similar concepts, such as task orientation (West and Anderson, 1996), climate for excellence (Eisenbeiss et al., 2008), task-related activities (Wang et al., 2016), task-oriented communication (Luor et al., 2010), and task-related team process (task-relevant information sharing, for example) (Braun et al., 2013), etc. These all highlight the team’s common-shared commitment to performing tasks and striving for excellence. In the early days, Tuckman (1965) defined task orientation as what a team needs to accomplish and how to accomplish it. Rousseau et al. (2006) adopted the concept of task-related collaborative behaviors, coupled with a specific and well-defined scope, including coordination, cooperation, and information exchange. They followed the action regulation theory, which suggests that individuals will undergo an execution phase after identifying the activities required to accomplish task goals (Frese and Zapf, 1994), and this concept is essentially similar to Tuckman’s definition. In addition, many scholars emphasize the importance of climate for excellence, which is a main construct of task orientation (Amabile et al., 1996; Anderson and West, 1998; Eisenbeiss et al., 2008). It focuses on team outcomes and reflects team-level attention to excellence rather than climate intensity (Schneider et al., 2002; Eisenbeiss et al., 2008).

Although scholars pay particular attention to different aspects when studying various team problems, the above task-oriented concepts have been emphasized for their significant role and positive impact on team management. Gray (2001) pointed out that participation in goal definition positively relates to project outcomes. Eisenbeiss et al. (2008) confirmed that teams with a high-level climate for excellence usually work harder and carefully choose the most promising strategies for goal achievement. However, regarding the boundary of task-oriented interaction, scholars have not reached a consensus. Ancona and Caldwell (1992b) held the view that internal task processes only have a positive impact on team-rated performance, and external communication only positively impacts managerial-rated performance. Van der Vegt and Bunderson (2005) suggested that external interaction has little impact on team performance jointly evaluated by jointly managers and followers, while Hülsheger et al. (2009) expressed a different result that both internal and external interactions are positive to team performance. Researchers have also become aware of the negative impact of task-oriented processes. For example, Janssen (2003) demonstrated that in a team with a high level of job involvement, innovative behavior is more likely to show its dark side, which highlights the latent cost of task-oriented processes. Another piece of evidence provided by Zhang and Bartol (2010) is that the relationship between creative process engagement and job performance shows an inverted U pattern. New insights into team research related to task-oriented processes are provided. Previous research may have overlooked the negative effects of task-oriented processes, resulting in conflicting results with purely linear models. However, this curvilinear pattern remains unexplored in dynamic teams—particularly regarding dynamic sports teams in digital environments, where fluid membership may amplify coordination costs (Bertolotti et al., 2015).

In practice, unlike the boundary-spanning interaction limited to leaders (Ancona and Caldwell, 1992b), all individuals can interact across team boundaries in open organizational networks. Thus, we do not distinguish between internal and external interactions in terms of task-oriented interactions. Regarding the factors that influence the relationship between task-oriented interaction and team organizing efficiency, this study focuses on team identification. Although other related processes may also affect their relationship, the moderation of team identification has been widely verified in many team processes (Wang and Rode, 2010; Bezrukova et al., 2009). Team identification is important in team research because it guides team members to respect team values and comply with team rules (Dukerich et al., 2002; Ashforth and Mael, 1989). With a high level of team identification, team members are motivated to take action to achieve team goals (Hogg et al., 2004). Empirical studies show that team identification enhances team-level outcomes (James and Greenberg, 1989; Worchel et al., 1998; Lin et al., 2017), supporting the above conceptualizations.

**Figure 1:**
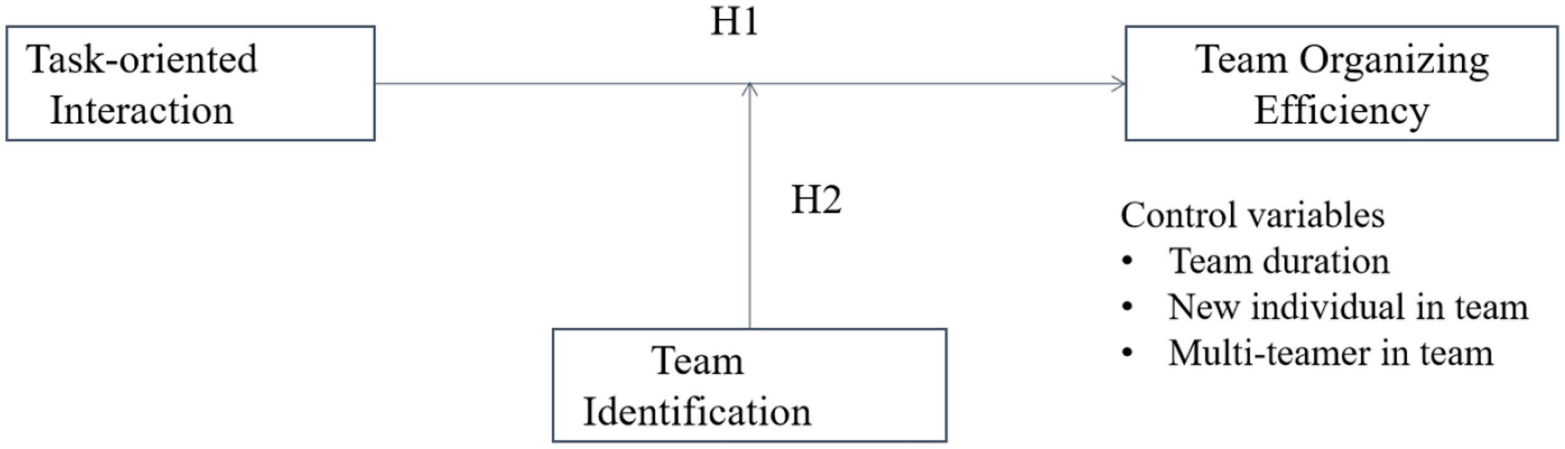
Theoretical model and hypotheses.

Despite the above discussions highlighting the link of task-oriented interaction and team identification to team organizing efficiency, how these constructs relate has not been specifically discussed, let alone in the digitally-mediated dynamic sports teams. In the following section, we integrate related team research to introduce our hypotheses concerning the direct and moderating effects of task-oriented interaction and team identification on team organizing efficiency. Our framework, presented in Figure 1, suggests that task-oriented interaction has an inverted-U impact on team organizing efficiency, and this impact is moderated by team identification.

## 3 Hypotheses development

### 3.1 Task-oriented interaction and team organizing efficiency

Team literature has broadly acknowledged the crucial role of task-oriented processes in team performance and many empirical studies have shown their evidence (Anderson and Potočnik, 2014; Braun et al., 2013; Rosing et al., 2011; Van Knippenberg and Sitkin, 2013). Team communication is taken as a positive predictor of team performance (Dionne et al., 2004), as individuals support each other by sharing task-oriented information and resources (Dirks and Skarlicki, 2009). Eisenbeiss et al. (2008) suggest that only in high-level task orientation, team innovation would be positively related to transformational leadership. Arnold (2013) found that effective horizontal communication promoted the dissemination of new practices in one organizational department, which could serve as benchmarks for improving positive employee attitudes and high performance in the organization elsewhere. Task-related activities, such as information elaboration, team learning, and team reflection, are also critical for team creativity (Hoever et al., 2012; Van der Vegt and Bunderson, 2005; Wang et al., 2016). However, a survey of air traffic controllers and pilots showed a difference that task-oriented communication did not have a direct influence on perceived work performance (Kang et al., 2017).

As we mentioned earlier, task-oriented interaction may have a potential cost, and Zhang and Bartol (2010) updated the acknowledgment by introducing attention capacity theory and activation theory (Kahneman, 1973; Gardner, 1986). Attention capacity theory proposes that limited attentional capacity and cognitive resources will be stimulated to produce arousal (Kahneman, 1973). Low arousal leads to low attention and effort, resulting in inadequate performance. However, when high arousal requires high cognitive resources, increased attention and effort cannot compensate for increasing challenges, leading to decrements in performance. Therefore, an inverted U-shaped pattern emerges. Activation theory expresses a similar phenomenon where high levels of activation challenge cognitive resources and lead to poor performance (Gardner, 1986). According to these theories and related results, we posit that task-oriented interaction will show a similar impact on team organizing efficiency. A low level of task-oriented interaction may reflect low activation toward team effort, while too high a level may represent a very high level of activation that leads to difficulties in meeting task demands. This circumstance will undermine team organizing efficiency.

Furthermore, team interaction practices in the dynamic organizational context that rely on collaborative technologies also support our view. Individuals are inherently beneficial for dynamic teams as crucial social resources (Mortensen and Haas, 2018). Interaction networks that extend beyond in-team individuals are advantageous for teams to achieve excellence (Ancona and Caldwell, 1992a; Sparrowe et al., 2001; Bercovitz and Feldman, 2011). Seeking external feedback can provide a higher level of supervisory appraisal and helps to make team improvements (Ancona, 1990; Ancona and Caldwell, 1992a). Interacting with individuals from different team backgrounds can also lead to the internalization of new information, ideas, knowledge, and resources, which may improve overall team performance (Wong, 2008; Oh et al., 2004). Especially in an open-boundary context, more interaction about the focus team generates higher arousal and leads the individual to select the focus team during multiple alternative choices.

However, teams should pay attention to its negative impact on outputs and psychological pressure on individuals (Ou and Davison, 2011; Zellmer-Bruhn, 2003; Taylor et al., 2008) when relying on collaborative technologies to support team coordination and interaction (Suh, 1999; Pazos et al., 2013). Collaborative technologies may cause disruptive interruptions and unwanted consequences (Garrett and Danziger, 2007; Rennecker and Godwin, 2005). Maintaining social interaction networks demands considerable effort and valuable resources, such as time and human resources (Day and Kilduff, 2003; Rodan and Galunic, 2004). As a result, excessive interaction may hinder team operation, increase coordination costs, and reduce team performance (Bertolotti et al., 2015). Thus, we hypothesize that:

*H1*: In digitally-mediated dynamic teams, task-oriented interaction has an inverted-U-shaped curvilinear relationship with team organizing efficiency.

### 3.2 Team identification as a moderator

Apart from the individual level, self-concept can also be defined at the relational or collective levels by relationships with pivotal others or the degree of feeling of belonging to the focus collective (Brickson, 2000; Lord et al., 1999). Identification with leader indicates the extent to which team leaders are considered part of the relational self of the focus follower (Van Knippenberg et al., 2004; Kark et al., 2003). Team identification is based on the collective self, indicating the extent to which members define themselves in terms of their memberships with the focus team (Dukerich et al., 2002; Ashforth and Mael, 1989; Hogg et al., 2004). High relational or collective self-concepts make individuals experience less distinction between their interests and those of focus others and focus teams (Andersen and Chen, 2002). Under high identification with leaders, members tend to be influenced by leaders and sensitive to the expectations of leaders (Van Knippenberg et al., 2004) and team leader’s needs are more likely to be considered by members (Sluss and Ashforth, 2007).

Team identification is often viewed as a motivator that reflects commitment at the team level (Van der Vegt and Bunderson, 2005). It is associated with positive team processes such as cooperation, positive behaviors, and attitudes (Hogg and Abrams, 1988; Hogg and Terry, 2000). High team identification also leads to various positive team-level outcomes, including organizational citizenship behavior (OCB) (Dukerich et al., 2002; Tyler and Blader, 2001), perceived performance, empowerment (Conger et al., 2000), team innovation (Liu and Phillips, 2011), efficacy, peer-directed voice (Liu et al., 2010), and organizational-based self-esteem (Kark et al., 2003). In this sense, high team identification may facilitate task-oriented interaction, enabling a focus on the common goal and increasing team organizing efficiency.

Critically, we argue that team identification plays a crucial moderating role in the curvilinear relationship between task-oriented interaction and team organizing efficiency in digitally-mediated dynamic context, where team identification can help the team overcome the challenges in terms of member collaboration and team coordination caused by fluid membership, geographic dispersion, and minimal formal structure (Wiesenfeld et al., 2001). Drawing on attention capacity and activation theories, high team identification mitigates the cognitive overload and coordination breakdowns typically associated with excessive task interaction. Shared identity, strong commitment to collective goals, and heightened trust facilitate spontaneous coordination, efficient information sharing, and constructive conflict resolution (even under high task load), thereby buffering the decline in organizing efficiency at higher levels of task interaction. Conversely, when team identification is low, teams lack this cohesive foundation. Members experience weaker commitment to team goals and are more susceptible to cognitive overload, friction in coordination, and inefficient conflict resolution as task interactions intensify. This exacerbates the negative effects at high task interaction levels. Furthermore, low team identification means members derive less inherent motivation and structure from their team affiliation, making the initial gain in efficiency from moderate task interaction less pronounced and the subsequent decline at high interaction more precipitous. Thus, the inverted-U relationship is expected to be significantly steeper under conditions of low team identification compared to high team identification.

Thus, we hypothesize:

*H2*: In digitally-mediated dynamic teams, team identification moderates the curvilinear relationship between task-oriented interaction and team organizing efficiency. The curvilinear relationship is more salient for teams with low-level team identification than teams with high-level team identification.

## 4 Empirical approach and data

### 4.1 Empirical context

We have been interested in team research for a long time. While in recent years, the organization form of teams has undergone tremendous changes. New requirements for team research are constantly proposed under multi-perspectives, such as dynamic social networks, multi-team members, teaming, subgrouping, etc. (Park et al., 2020; Mortensen and Haas, 2018). As a result, it is difficult for traditional methods, such as randomized experiments, and even questionnaire surveys based on real scenes, to reproduce the real and complex organizational environment. To support the long-term tracking and in-depth study of dynamic teams in a complex, interlacing, and multi-level organizational network, our research group has been developing an O2O community based on real-world organizational scenarios, creating a digitally mediated, dynamic, and boundary-spanning environment for amateur football self-organizing.

#### 4.1.1 Scenario setting

The scenario to be platformed should meet certain conditions. First, the organizational units in the scenario fit the general definition of “team,” including people, relations, interaction, responsibility, goals, and so on (Kozlowski and Ilgen, 2006). Second, the organizational scenario should be clear and simple to be platformed online and to track related data of teams and individuals. Finally, after being platformed, the online scenario needs to meet the characteristics of new forms, such as dynamic, social networking, multi-teamers, fuzzy boundaries, etc. (Mortensen and Haas, 2018). Our research group took the amateur soccer kickers as the object and chose their game (i.e., soccer activity) organizing as the scenario to be platformed. This context satisfies the foregoing three conditions well. In addition, there are precedents for discussion and research of sports teams, such as football teams (Kozlowski and Klein, 2000; Dukerich et al., 2002; Myers et al., 2004) and baseball clubs (Kozlowski and Klein, 2000), which have proved that the empirical results of similar scenes can be popularized and applied in traditional team management.

#### 4.1.2 Platform design

*Basic setting and R&D environment*: with the internet plus technologies, we have realized an O2O community of the activity organizing and team management scene of amateur soccer. It is a WeChat applet, called DaZhiYouQiu (DaZhi hereafter), and aims to improve the entire efficiency of activity organizing of amateur soccer. The applet is easier to develop than mobile applications, especially in matching different mobile operating systems such as Android and IOS. Besides, with the dissemination in the WeChat ecosystem, such as WeChat groups and Moments (see Appendix A), DaZhi could be promoted and applied more quickly and easily as WeChat had over 1 billion active users per month (Tencent, 2018).

*DaZhi initially launched in April 2018*: it encourages users to organize publicly visible activities, making it easier for individuals to find suitable activities. In turn, these individuals seeking suitable activities help to decrease activity miscarriage. Fortunately, with our continuous optimization and promotion, DaZhi became popular among amateur soccer players and has over 970,000 users with about 1,000 new users per day (Please refer to Appendix B).

**Figure 2:**
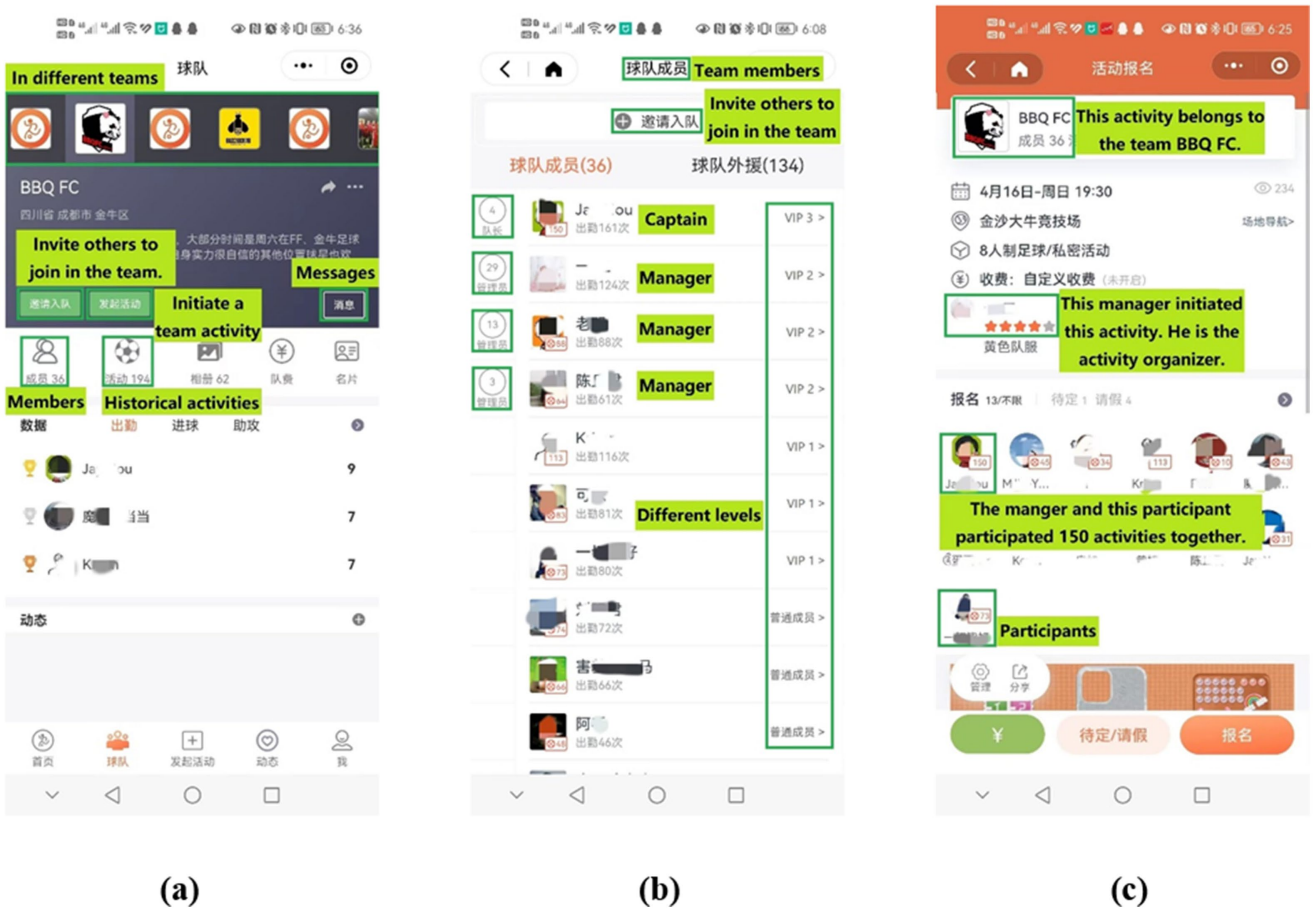
Multi-layered organizational features from team management perspective. **(a)** Cross-team management interface, **(b)** member role configuration, **(c)** activity-based connection mechanism.

*Settings for teams in DaZhi:* users form teams freely and can invite more people to join their teams and activities. Teams exist as dynamic centers of participants (namely, members). Activity organizing is the basic team task. Members’ shared desire, to participate in activities, is consistent with team goals. In a team, members have different roles, responsibilities, and divisions of labor, such as team manager, team member, organizer(s) of activities, activity participants, etc. Membership is changeable, and multi-teamers exist in DaZhi. Online interactions among members are available. As organizational units, teams relate to each other through shared members or indirect interactions between their members. Thus, different kinds of links have built a complex social organizational network for amateur kickers (see Figure 2).

#### 4.1.3 Description of key variables

DaZhi has become an increasingly complex and comprehensive applet over a hundred iterations. Here, we will not delve too much into the applet itself but only discuss the relevant content of this study.

*Team organizing efficiency:* following Input-Process-Output (IPO) models of team functioning (Kozlowski and Ilgen, 2006), we define team organizing efficiency as the ratio of outputs (e.g., viable participation levels achieved) to inputs (e.g., organizational effort, time, resources consumed) in executing the core organizing task. In the context of dynamic, self-organizing teams like amateur football collectives, successfully mobilizing sufficient participants (output) within temporal and coordinative constraints represents a critical indicator of organizing efficiency (Wang et al., 2021; Jiang et al., 2022; Shvets et al., 2024). We operationalize this core team-level capability as the average number of participants per successfully organized activity over the observation window. This metric directly captures the realized output (participants mobilized) relative to the fundamental organizing input goal (achieving a viable activity) repeated multiple times during the period. In this specific context, higher average participation signifies greater efficiency in converting organizing inputs (planning, communication efforts, platform use time) into successful outputs (meeting or exceeding minimum viable group size requirements), particularly when accounting for fixed organizing time constraints per activity (typically 2–3 days setup on the platform) (Wang et al., 2021; Jiang et al., 2022). While alternative efficiency metrics (e.g., resources used per participant) are theoretically possible, average realized participation is a well-established, outcome-focused indicator for organizing efficiency in settings reliant on voluntary engagement and self-coordination (Shvets et al., 2024; Ma et al., 2023).

In amateur football teams operating within a dynamic platform context, a core organizing challenge is reliably assembling a viable group for each activity against coordination constraints (e.g., time, venue, competing commitments). Team organizing efficiency therefore captures the team’s ability to convert its organizing efforts into achieved participation, reflecting the output (actual participants recruited) relative to the fundamental input goal of meeting minimum viable squad size. Following this logic and the constraints observed on the platform (teams typically require 2–3 days to organize an activity), we operationalize it as the average number of participants per successfully organized activity over the observation window. This metric directly reflects the realized output per organizing attempt in this specific context, where achieving higher average participation with limited organizing time and resources signifies greater efficiency.

*Team organizing efficiency:* according to the organizational scenario, the execution task of an amateur soccer team is to complete the organization of team activities, including determining the location, publishing the online post for the activity, and involving enough individuals in the activity. In an activity, participants naturally form a group, and the group size is the outcome of this activity’s organization. The average outcome of a team during a period is taken as the team’s organizing efficiency.

*Task-oriented interaction:* different kinds of interactions exist in teams, and this study focuses on task-oriented interactions. We designed a unique function as task-oriented interaction, i.e., the “Share” button on the activity page. Through this button, an activity can be shared with WeChat friends, groups, and moments to invite people to participate in the activity. With the event analysis tool of the WeChat Public Platform, we can easily capture the using data of the “Share” button. Second, this button can distinguish the activity sharing from other sharing, for example, the report of the applet (see Appendix A). The “Share” function operationalizes task-oriented interaction because it enforces goal-directed resource coordination (Rousseau et al., 2006) – a dominant form of task execution in boundary-open teams (Mortensen and Haas, 2018).

*Team identification* is built on the “Like” function on the personal page. Team identification refers to the number of team members who mark “Like” on the team leader(s)’ personal page. Essentially, it is an identification with team leader. While in our context, it can be applied to team identification based on two points. First, in contrast to fluid team members and changing teams, team leaders are relatively stable in amateur soccer teams and can serve as centers of change. Therefore, team identification is practically close to the identification with leader. Secondly, the attention on the team itself often goes beyond the scope of team identification. For example, external attentions from other teams usually show their interest in having a match with the focus team. For distinction, we have designed a message board on the team homepage for expressing external interest (see Appendix C). Meanwhile, we designed related functions to enrich the overall concept of team identification. For more details, please refer to Appendix D.

#### 4.1.4 Control variables

*Team duration:* we have recorded the time when a team is created, to calculate team duration. Considering that the longer a team operates, the more experienced the team may be in activity organizing and team management.

In addition, we introduce another two variables in terms of the dynamicity and open boundary in the community. New individual in team is the proportion of new individuals, measuring the team’s dynamic degree from the time of the vertical dimension. By comparing team members from two consecutive periods, we can determine how many new individuals have joined the team. It represents the level of the team’s openness, that is, to what extent new individuals are recruited to the team. Multi-teamer in team is the proportion of multi-teamers in a team, measuring the team’s dynamic degree in terms of dynamic connections with other teams horizontally. By comparing the focus team with other teams, we can determine how many shared members (multi-teamers) are in the focus team (Table 1).

**Table 1:** The mapping table of theoretical variables—observed indicators–data sources.

Construct	Operationalization	Data source	Transformation
Team organizing efficiency	Avg. participants per activity	The “activity_member.csv” file exported from the backend database of DaZhi	Team-level monthly mean
Task-oriented interaction	Share button click count	Event analysis tool of the WeChat Public Platform	Monthly sum per team
Team identification	Likes to leader’s profile	The “attention.csv” file exported from the backend database of DaZhi	Count of likes of in-team members to team leaders
Team duration	Days since team creation	The “team.csv” file exported from the backend database of DaZhi	Observation date - Creation date
New individual in team	Proportion of new members between t-1 and t	The “team_member.csv” file exported from the backend database of DaZhi	New_members_t /Total_members_t
Multi-teamer in team	Proportion of members in >1 team	The “team_member.csv” file exported from the backend database of DaZhi	Number_of_Members_in_multiple_teams/Total_members_in_the_focus_team

“t” represents the observation window and “t-1” represents the immediately preceding time window of the same length as the observation period.

### 4.2 Dataset

Our research period is set to 1 month, synthetically considering the organizational habits of amateur soccer activities and the dynamic forms. About half teams on the platform organize an activity every week. If the period was 1 week, the data observed would undergo strong uncertainty interference. For example, there would be not enough people participating in one activity of a company team due to temporary overtime work in a week. Or at some special times, such as team annual meetings, participants might far exceed the usual (see Appendix E). Therefore, a longer observation period is necessary. However, if it was too long, for example, 1 year, the team’s dynamic nature would be overlooked. In practice, due to family, work, and physical factors, amateur soccer teams change frequently, and on average, teams have a quarter of membership changes per month, including leaving and joining.

Data were extracted from the DaZhi platform during November 2020, capturing 2,215 published activities. We applied sequential filters to ensure ecological validity. Firstly, 386 activities were excluded as either canceled (indicating organizational failure) or having ≤4 participants (below the 5v5 minimum squad requirement for meaningful football matches). Secondly, the remaining 1,829 activities were then filtered by team affiliation. We eliminated activities lacking team associations (since our unit of analysis requires team-level organizational processes), and removed teams organizing <4 activities during the observation window. This frequency threshold (≥4 activities/month) was established based on standard amateur football cycles, where teams maintaining at least weekly matches (>1 activity/week) demonstrate the stable organizational patterns and sustained coordination capabilities central to our investigation. Conversely, lower frequencies indicate irregular operations and transient social aggregation rather than persistent team dynamics relevant to organizational learning and effectiveness. Furthermore, organizational failure, such as the inability to organize activities, often reflects underlying issues in team processes, including poor coordination, lack of clear direction, and ineffective leadership. These deficiencies are strongly associated with reduced group performance and the inability to achieve collective goals, making such teams unrepresentative of viable, functioning collectives (Zaccaro and Klimoski, 2002). Therefore, including teams that fail to organize activities could introduce noise and bias, as their performance does not reflect the dynamics of functioning teams. Excluding them ensures that analyses focus on groups with the potential for meaningful participation and organizational learning, thereby increasing the validity and relevance of research findings (Gong et al., 2020). The final sample comprised 214 teams with 1,204 activities, encompassing 5,923 individuals and >6,000 team-member relationships, collectively ensuring both theoretical alignment with dynamic team research frameworks (Mortensen and Haas, 2018) and statistical robustness. The final sample comprised 214 teams and 1,204 associated activities, encompassing 5,923 unique individuals and >6,000 team-member affiliations. Analysis revealed substantial heterogeneity across two dimensions:

1. Activity frequency: teams organized 5.62 activities on average (SD = 5.93), stratified as high-activity (>6 activities; 15.9% of teams), medium-activity (5–6 activities; 25.7%), and low-activity (exactly 4 activities; 58.4%);
2. Geographic distribution: teams spanned 19 urban centers across 12 Chinese provincial-level administrative divisions and Japan, exhibiting significant spatial clustering—notably in Beijing (34.58% of sample), Shanghai (10.28%), and Tianjin (9.81%), with secondary concentrations in Sichuan (8.88%) and Jiangsu (2.80%). Location data were unavailable for 23 teams (10.75%).

## 5 Results

### 5.1 Descriptive statistical analysis

Table 2 shows descriptive statistics. Team organizing efficiency significantly relates to task-oriented interaction (r = 0.218, *p =* 0.001) and team identification (r = 0.181, *p =* 0.008), which means that team organizing efficiency might be explained by them. The tolerance test indicates no potential threat of multicollinearity as values of variance inflation factor (VIF) range from 1.076 to 2.212 (Tan and Chen, 2022).

**Table 2:** Descriptive statistics: means, standard deviations, and correlations of the variables.

Variables	Mean	SD	1	2	3	4	5
1. Team organizing efficiency	12.616	3.524					
2. Task-oriented interaction	51.692	35.844	0.218^**^				
3. Team identification	1.888	3.019	0.181^**^	0.070			
4. Team duration	212.846	161.404	−0.004	0.095^	0.396^***^		
5. New individual in team	0.226	0.243	−0.028	0.293^***^	−0.158^*^	−0.295^***^	
6. Multi-teamer in team	0.070	0.097	−0.120^*^	0.214^**^	0.147^*^	0.166^**^	0.151^*^

A total of 214 teams related to 5,923 individuals generated 6,216 individual-team relations with 1,204 activities, which implies that multiple team memberships existed. SD, standard deviation. ^*p <* 0.1, *p < 0.05, **p < 0.01, ***p < 0.001.

### 5.2 Hypotheses testing

We performed a stepwise regression method to run the empirical model and examined the incremental contributions for each step, and all variables were standardized using Z-score normalization prior to model validation. Table 3 demonstrates the results. Specifically, Model 1 indicates that control variables are not significant predictors of team organizing efficiency. Model 2 implies that the linear, main effect of task-oriented interaction is significantly positive (*β* = 0.999, *p <* 0.001) with a significant incremental contribution (ΔR^2^ = 0.069, *p <* 0.001). When the quadratic term is concerned in Model 3, the incremental variance (ΔR^2^ = 0.026, *p =* 0.015) is significant. The coefficient for the quadratic term is significantly negative (*β* = −0.274, *p =* 0.015), and for the linear term is positively significant (*β* = 1.543, *p <* 0.001), indicating an inverted-U-shape effect of task-oriented interaction on team organizing efficiency.

**Table 3:** Regression results of team identification moderation on task-oriented interaction and team organizing efficiency.

Variables	Model 1	Model 2	Model 3	Model 4
Team duration	0.051	−0.115	−0.149	−0.377
New individual in team	−0.019	−0.339	−0.297	−0.259
Multi-teamer in team	−0.428^	−0.566^*^	−0.457^^^	−0.484^*^
Task-oriented interaction		0.999^***^	1.543^***^	1.425 ^***^
Team identification				0.400
TOI^2^			−0.274^*^	−0.275^*^
TOI × TI				−0.837^*^
TOI^2^ × TI				0.501^**^
*R^2^*	0.015	0.084	0.110	0.178
*ΔR^2^*	0.015	0.069^***^	0.026^*^	0.068^**^

Coefficients are unstandardized. Maximum VIF = 2.212, which is much lower than 10. DV, team organizing efficiency. TOI, task-oriented interaction; TI, team identification. ^*p <* 0.1, *p < 0.05, **p < 0.01, ***p < 0.001.

The inflection point occurs at a standardized task-oriented interaction value of 2.82 (z-score), corresponding to a raw value of 152.62. This value, while exceeding the sample mean, remains within the empirically observed range (min = 5, max = 264) and is substantively meaningful for two reasons: (1) as per Haans et al. (2016) criteria for testing inverted U-shaped relationships, our model satisfies the critical conditions; (2) approximately 5.61% of teams (*n* = 12) exhibited interaction levels ≥152, with 5 teams exceeding 180–indicating these represent behaviorally significant cases of intensive coordination rather than statistical outliers.

Therefore, H1 is supported. The positive linear effect conforms to the previous findings that task-oriented interaction contributes to the achievement of team tasks and goals. However, further insights are given when the quadric term is included. The positive effect exhibits diminishing returns and ultimately becomes negative when task-oriented interaction increases over a certain high degree. This indicates that task-oriented interaction will not show its dark side until it ascends to a certain point.

**Figure 3:**
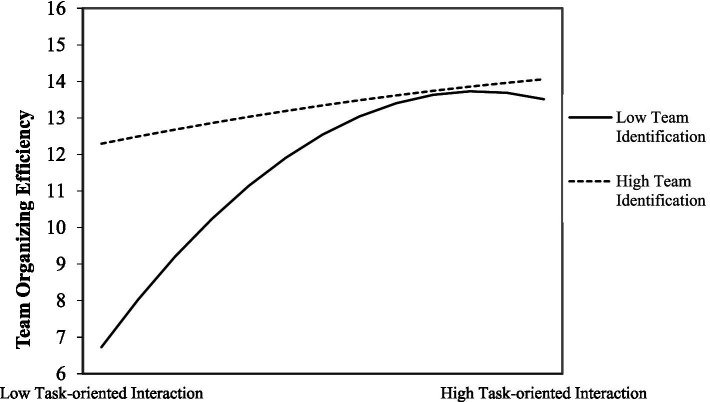
The curvilinear moderation of team identification on task-oriented interaction and organizing efficiency.

The second hypothesis emphasizes the moderation between task-oriented interaction and team identification. To this end, we introduce regression models with moderating effects (Shafique and Naz, 2023). The full regression (Model 4) demonstrates the additional contribution of team identification’s moderation effects on the relationship between task-oriented interaction and team organizing efficiency (ΔR^2^ = 0.068, *p =* 0.001). Team identification negatively interacts with task-oriented interaction (*β* = −0.837, *p =* 0.017) while significantly and positively interacts with its quadratic term (*β* = 0.501, *p =* 0.009), suggesting that higher team identification flattens the inverted-U-shape relationship between task-oriented interaction and team organizing efficiency (Haans et al., 2016). The results confirm our theoretical reasoning that team identification benefits the effects manifested by other team processes. For better illustration, we follow the advice of Dawson (2014) and plot Figure 3, which shows the considerable difference in task-oriented interaction’s impact on team organizing efficiency in terms of team identification. Thus, H2 is supported. Figure 3 indicates that, teams with high identification do not have the inverted-U pattern as they do not exhibit any decreasing return to scale in terms of team organizing efficiency. However, when team identification is low, task-oriented interaction demonstrates a clear inverted-U-shaped pattern (*β* = −0.583, *p =* 0.009), and the inflection point occurs at the task-oriented interaction value of 1.05, which corresponds to an actual task-oriented interaction value of 89.33. This inflection point is significantly smaller than the inflection point of Model 3, indicating that the entire inverted U-shaped curve graph is significantly shifted to the left. The moderation demonstrates a deeper understanding of the pattern in which teams with low identification will display decreasing returns at a certain point. After that, increasing task-oriented interaction will soon be a concern for these teams and their members.

### 5.3 Robustness testing

To confirm the quadratic pattern among task-oriented interaction and team organizing efficiency, we have conducted several robustness checks, following (Assaf and Tsionas, 2019). We tested exponential, logarithmic, and cubic relationships, and no specification increased the model fit. Additionally, we checked Cook distance (Cook, 1979), and the values range from 0 to 0.138, much lower than 1, the cut-off value, indicating no potential threat of extreme observations. Furthermore, to test the robustness of the general findings, we repeated the entire study with another dataset collected from DaZhi in May 2021. The two-Sample Kolmogorov–Smirnov test indicates a significant difference between this dataset and the previous one (Zaiontz, 2020). The results support the inverted-U relationship between task-oriented interaction and team organizing efficiency and the curvilinear moderation of team identification (see Appendix F).

## 6 General discussion

Our study provides support for the inverted-U impact of task-oriented interaction on team organizing efficiency in digitally-mediated dynamic teams—a pattern empirically validated through evidence from amateur sports organizing, where fluid membership, voluntary participation, and platform-dependent coordination characterize the context. Especially, our findings indicate a positive relationship between task-oriented interaction and team organizing efficiency up to a certain point. Higher levels of task-oriented interactions relate to a decrease in team organizing efficiency once beyond this point in these loosely-coupled systems. This finding conforms to activation theory, which holds that moderate stimulating is beneficial while over-stimulating brings loss (Gardner, 1986). In the current context, when stimulated by task-oriented interactions, teams will engage in organizing activities, and team organizing efficiency increases with the rising of task-oriented interaction up to a certain point. However, beyond that point, the stimulation and the demand for the team is exorbitant, resulting in a high level of pressure and responsibility, and ultimately leading to a decline in team organizing efficiency.

Moreover, we found that team identification moderated the inverted-U pattern. Crucially, in boundary-open teams where member turnover and geographic dispersion are normative, strong identification functioned as a digital buffer. The evidence from digitally-mediated amateur sports teams suggests that with low team identification, teams followed the inverted-U pattern between task-oriented interaction and team organizing efficiency. In contrast, with high team identification, teams displayed relatively high levels of team organizing efficiency regardless of the level of task-oriented interaction. This finding highlights the importance of team identification in mitigating the negative effects of excessive task-oriented interaction on team organizing efficiency, providing new insights into their interplay in digitally-mediated dynamic teams.

The empirical validation of this study was conducted in amateur sports organizing contexts, where dynamic teams operate through digitally-mediated coordination in informal environments. This setting serves as a critical testing ground for theorizing efficiency dynamics in digitally-mediated dynamic teams. Consequently, our findings affirm the model’s validity for digitally-mediated dynamic teams while they caution against direct extrapolation to high-interdependence teams requiring physical co-creation (e.g., surgical teams, product design units), as their coordination relies on synchronous, resource-intensive collaboration distinct from the lightweight broadcasting paradigm examined here.

### 6.1 Theoretical contributions

Our study extends dynamic team literature by revealing the curvilinear role of task-oriented interaction—particularly in digitally-mediated sports organizing contexts—with team identification serving as a pivotal moderator. This study provides contributions to the dynamic sports team research as follows.

First, by applying activation theory (Gardner, 1986), we shed light on the inverted-U impact of task-oriented on team organizing efficiency in the digitally-mediated teams. Our results suggest that a non-linear pattern may be a more accurate way to explain the inconsistent findings in previous studies on task-related processes (Hülsheger et al., 2009; Kang et al., 2017; Peltokorpi and Hasu, 2016). For example, previous studies have reported insignificant effects of task-oriented processes on outcomes (Ancona and Caldwell, 1992b; Van der Vegt and Bunderson, 2005), probably because the different effects of different levels of task-oriented processes cancel out each other. Our study underscores the importance of identifying the optimal level of task-oriented interaction to achieve maximum team organizing efficiency in the context. This context is characterized by greater uncertainty, resource constraints, and interdependence than traditional organizational settings, making interactions more complex. Our evidence confirms its power to explain digitally-mediated organizing fragility.

Second, this study contributes to team identification literature. Our findings indicate the good application of social identity theory in digitally-mediated teams, and imply that moderate task-oriented interaction may be inadequate to rouse organizing efficiency in these contexts. The results highlight the critical role of team identification in moderating the curvilinear relationship between task-oriented interaction and team organizing efficiency. This might add another layer of explanation to previous studies that have reported inconsistent conclusions on the relationships between task-oriented processes and team outcomes. Our study suggests that moderators such as team identification categorize teams into different groups, within which task-oriented processes can lead to distinct outcomes. With the aid of team identification, task-oriented interaction can function as an efficiency driver and avoid diminishing returns. Our findings conform to previous research (Bezrukova et al., 2009; Tyler and Blader, 2001), which suggests the positive role of team identification. That is, task-oriented interaction can be a powerful tool for team cooperation, coordination, and task completion within the context of a strong identification. Extant research has shown that team identification is an important predictor of team outcomes (Olkkonen and Lipponen, 2006; Dukerich et al., 2002). By employing team identification as a moderator, our study contributes to the prediction of the curvilinear relationship between task-oriented interaction and team organizing efficiency.

Third, amateur sports platforms validate non-workplace digitally-mediated organizing principles. Their autonomy, fluidity, and tech-dependence mirror emerging work forms, probably making them ideal microcosms for post-bureaucratic organizing research. Our findings based on digitally-mediated teams in non-workplace contexts are consistent with those of the workplace, which indicates that despite the differences in organizational objects, groups, and environments, there are similarities in individuals’ attitudes and behaviors concerning teams (Costa, 2019; Dirks, 2000). Therefore, while observed in sports contexts, the dynamics of diminishing returns may generalize to other digitally-mediated dynamic teams.

In summary, our study suggests that traditional team management theories and practices can be expanded to digitally-mediated dynamic teams. In this context, individuals have greater fluidity and flexibility in choosing different teams, which may facilitate the discovery of the curvilinear impact of task-oriented interaction on team organizing efficiency. Specially, under the psychological pressures and tensions caused by the strong demands and stimulation of the focus team, individuals can choose to avoid these pressures and tensions, such as temporarily transferring to activities organized by another team. As a result, the focus team is more likely to exhibit a decline in organizing efficiency. Furthermore, our study highlights that the turning point of the curve is at a relatively high level of task-oriented interaction, which emphasizes the need to consider the costs associated with excessive task-oriented processes, particularly in a dynamic and open-boundary organizational context, where the risk of turnover is high, and individuals may have a lower tolerance for such processes. It is important to note that traditional team structures that are more stable may foster higher team cognition, leading to greater team identification and ultimately, higher team organizing efficiency. This may explain why previous studies, which mostly rely on stable teams, have not uncovered the negative effects of excessive task-oriented processes.

### 6.2 Practical implications

Our field study has important practical implications for amateur teams’ management in digitally-mediated contexts. Firstly, our findings enlighten to monitor interaction thresholds. Team leaders should consider the diminishing return of task-oriented interaction in conjunction with effort allocation and the practical requirements of coordination and cooperation within a team. Thus, teams can determine the optimal point at which to invest in task-oriented interaction thereby maximizing team organizing efficiency. Our results suggest that when teams over-spend on task-oriented interaction, it may simply increase the team’s input but lead to diminishing returns, resulting in a decrease in team organizing efficiency. In general, team members value their interactions. This support from members reinforces team-process-related theories, in that members attach considerable importance to interactions and tolerate the increase until they reach a certain level. Therefore, team leaders in sports platforms are advised to track interaction volume and set alerts to preempt notification storms. While low-to-moderate levels of task-oriented interactions can increase team organizing efficiency, team leaders should monitor the team’s threshold of tolerance for such interactions and carefully assess if the level of task-oriented interaction has reached its optimum level and make appropriate interventions to increase or decrease it when necessary. Beyond the turning point, the increase in task-oriented interaction can lead to physical, psychological, and emotional strains and pressures, making it challenging for teams to cope with demanding demands. In this circumstance, teams need to provide support for individuals and help them focus on how to cope with these strains and pressures.

Secondly, teams should actively develop team identification by improving members’ sense of belonging to and enhancing their perceived support from the team (Wiesenfeld et al., 2001). Team identification can be developed through organizational or training interventions (Pratt, 1998; Wiesenfeld et al., 2001). Team leaders should be knowledgeable about team identification very well and develop guidelines and activities to enhance individuals’ psychological resources. Although the cost of enhancing team identification may be high, it potentially leads to improvements in team organizing efficiency. As our findings suggest, teams with a relatively high level of team identification reported superior team organizing efficiency. Enhanced team identification may further mitigate the diminishing returns associated with excessive task-oriented interaction. Therefore, team leaders should prioritize cultivating team identification alongside promoting task-oriented practices, ultimately fostering a more productive work environment.

Thirdly, similar organizational communities and platforms should support to engineer digital identification for teams, in order to help managers better manage their teams, stimulate team vitality, and promote the sustainable development of the community. We suggest that the platform set up relevant features to enhance team identification, such as curating team symbols (e.g., giving team members achievement badges from the team), algorithmically highlight mutual dependencies (e.g., displaying members’ shared data and effort achievements), gamifying collective milestones (e.g., 100 activities organized successfully), etc.

### 6.3 Limitations and future research

As is the case with all studies, our study has limitations. Firstly, we only examine one construct of task-oriented processes, i.e., task-oriented interaction. Future research could contribute to the literature by discussing the impact of other task-oriented processes, such as task-related collaborative behaviors and other task-related activities.

Secondly, we examined team identification’s curvilinear moderation effect on the relationship between task-oriented interaction and team organizing efficiency. However, the figures of the main test (Figure 3) and robust test (Appendix Figure F1) showed a significant difference, which suggests the possibility of other moderating factors that may affect the relationship between task-oriented interaction and team organizing efficiency. Future research could investigate the role of team cohesion as another potential moderator in this relationship, as team cohesion has been shown to have a moderating effect in prior team literature (Van Knippenberg and Sitkin, 2013). A more comprehensive model that considers multiple moderators may provide a more complete understanding of the complex dynamics involved in task-oriented processes and team performance.

Furthermore, our study was conducted in a specific context, which may limit the generalizability of our findings. Future research could expand the investigation to other contexts, such as different industries or types of work environments, to test the generalizability of our results. Finally, our study utilized a cross-sectional design, which may limit causal inference. Future research could adopt longitudinal designs to establish causal relationships between task-oriented processes, team identification, and team organizing efficiency.

## 7 Conclusion

Our study contributes to dynamic team literature by integrating task-oriented processes and team research, proposing a theoretical model with hypotheses related to team identification’s curvilinear moderation on task-oriented interaction and team organizing efficiency in digitally-mediated dynamic teams—contexts defined by fluid membership, voluntary participation, and platform-dependent coordination. To test hypotheses, we designed a field experiment using a platform for organizing amateur football activities—a boundary-open digitally-mediated organizing archetype. Participants freely create teams, manage members, and organize activities through this platform. By tracking behavioral traces from 455 spontaneous sports teams, we validated our model while overcoming ecological validity limitations of traditional experiments (Hauser et al., 2017). Additionally, the real organizational scenario provides more robust causal-effect tests and makes our data and analyses more objective and convincing (Hughes et al., 2018).

Overall, findings reveal that task-oriented interaction exhibits an inverted-U effect on efficiency in these volatile digital contexts, and team identification moderates this relationship. Our study highlights the importance of balancing task-oriented interaction with other team processes and developing team identification to optimize team efficiency—strategies essential for sustaining efficiency in boundary-open teams and analogous digital collectives. Future research could expand our findings by investigating other task-oriented processes and potential moderators and examining our model in other different organizational contexts to refine digitally-mediated organizing theory.

## Data Availability

The raw data supporting the conclusions of this article will be made available by the authors, without undue reservation.
