# Supplementary material for: Diminishing returns of task-oriented interaction in digitally-mediated dynamic teams: evidence from amateur sports organizing
Source: Front Psychol. 2025 Aug 18;16:1548846. doi: 10.3389/fpsyg.2025.1548846 (PMC12400965; doi:10.3389/fpsyg.2025.1548846)
Supplement: Supplementary file 1 [file Data_Sheet_1.docx]

Diminishing Returns of Task-Oriented Interaction in Digitally-Mediated Dynamic Teams: Evidence from Amateur Sports Organizing

# Appendix A: DaZhiYouQiu and Its Promotions in WeChat

An example of sharing the applet. Figure A1 shows how to initiate the sharing of DaZhiYouQiu, and Figure A2 displays the sharing screenshot in a WeChat group.


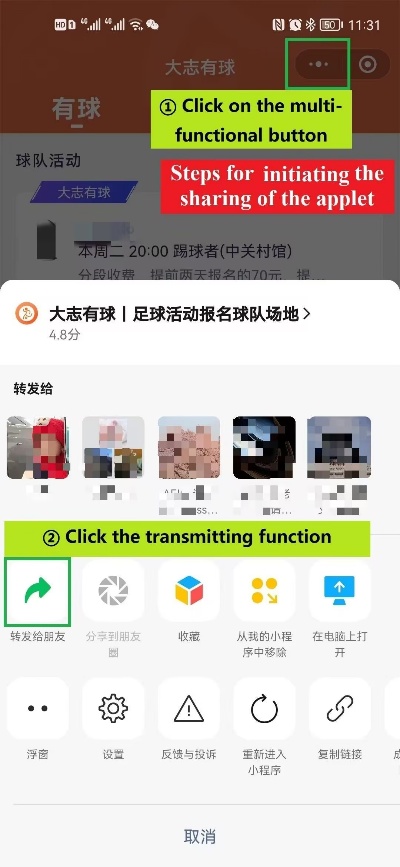


Figure A1. Initiate the sharing of the applet.


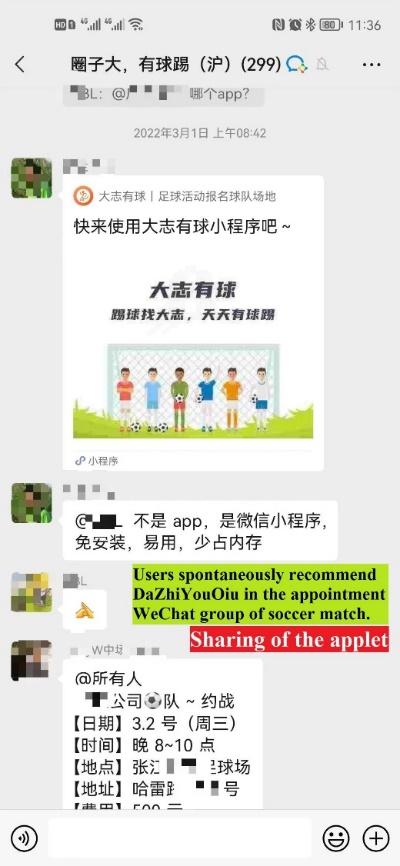


Figure A2. The applet is shared in a WeChat group.

The following pictures demonstrate an example of sharing an activity from DaZhiYouQiu to a WeChat group and one's WeChat moments. Figure A3 shows how to initiate the activity sharing in DaZhiYouQiu, and Figure A4 and A5 display the sharing screenshots in WeChat groups and Moments.


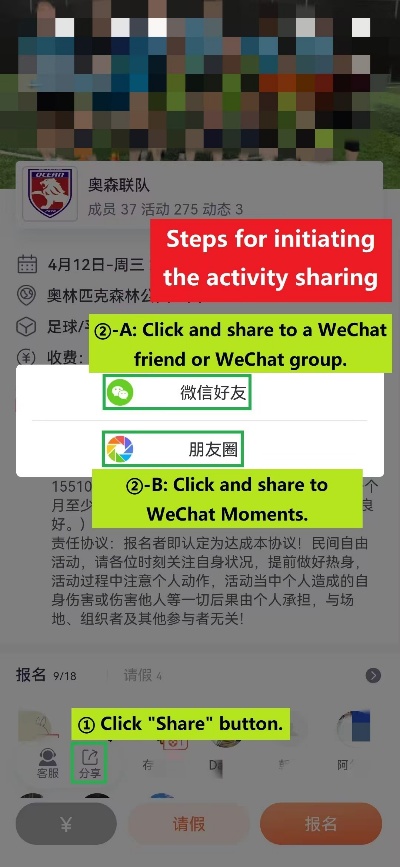


Figure A3. Initiate the sharing of the activity in DaZhiYouQiu.


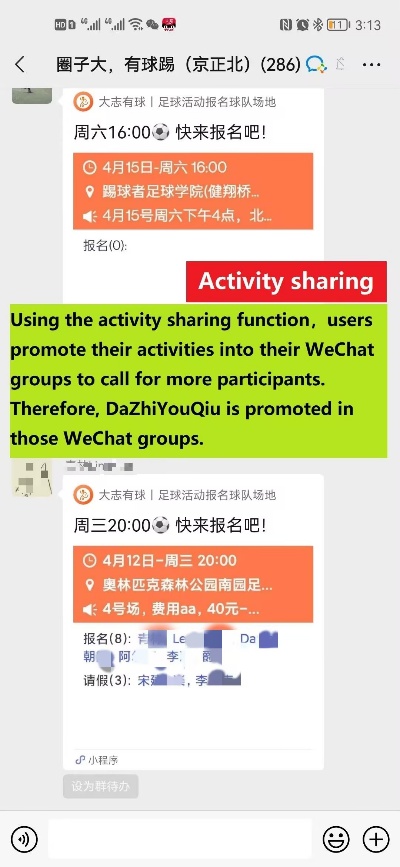


Figure A4. The activity in DaZhiYouQiu is shared in a WeChat group.


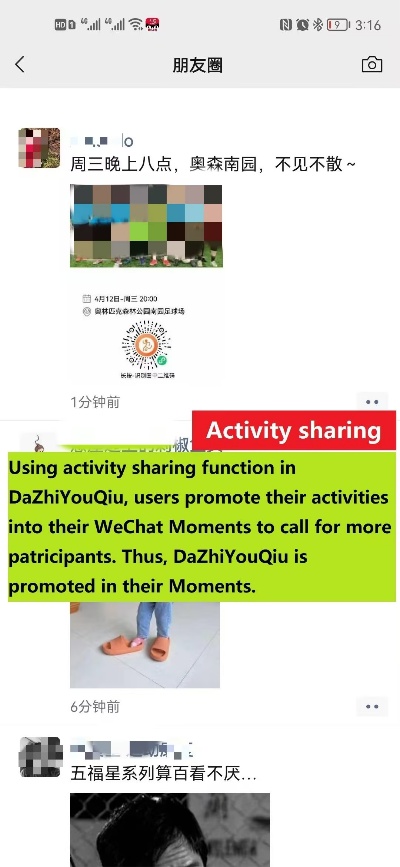


Figure A5. The activity in DaZhiYouQiu is shared in one's WeChat group.

# Appendix B: DaZhiYouQiu's Growing User Base and Scoring – Evidence from Aladdin Mini Program, A Third-Party Intelligent Analysis Platform for Applets

The statistics in Figure B1 was updated at 15:31 pm, April 10, 2023. They are hourly-updated and can be accessed by authorized personnels. The statistics in Figure B2 were updated on 8 am, April 10, 2023. Aladdin index refers to the comprehensive evaluation according to the previous day's usage, and is updated once a day. Everyone can access to the scoring and Aladdin index as long as the applet has been registered in Aladdin.


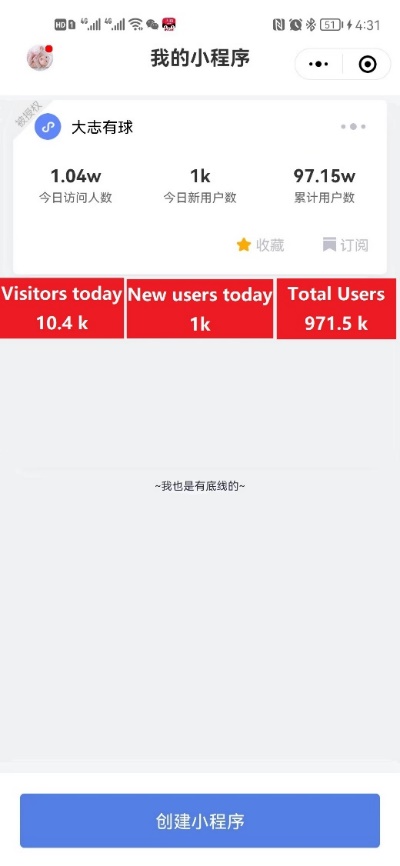


Figure B1. User data of DaZhiYouQiu – a screen from Aladdin mini program.


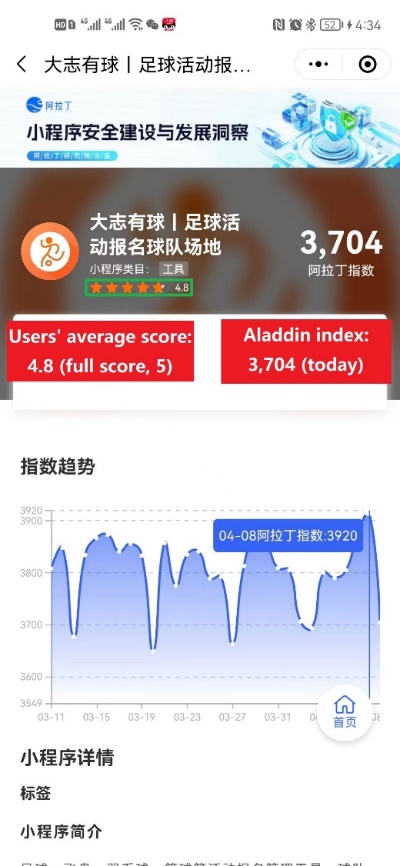


Figure B2. User score and Aladdin index of DaZhiYouQiu – a screenshot from Aladdin mini program.

# Appendix C: Teams, Activities, and Different Roles

Screenshots of a team and its members are shown from the perspective of a team manager. Figure C1, the first screenshot, is the homepage of the team BBQ FC, and the top navigation bar shows that this manager is in different teams simultaneously. Figure C2, the second screenshot, is the member list of BBQ FC, indicating that members have different roles in the team, such as captain, manager, and other members. Note that, the manager may have different other roles in different teams.

Figure C3, the third screenshot, is a team activity. In the activity, two roles are included, organizer and participant. The manager initiated this activity and thus he is the activity organizer. The other users are participants. Users are connected through common activities. Teams are connected though users who have participated some activities together or users who are multi-teamers (in different teams simultaneously). Thus, users and teams are nested in a complex organizing environment.


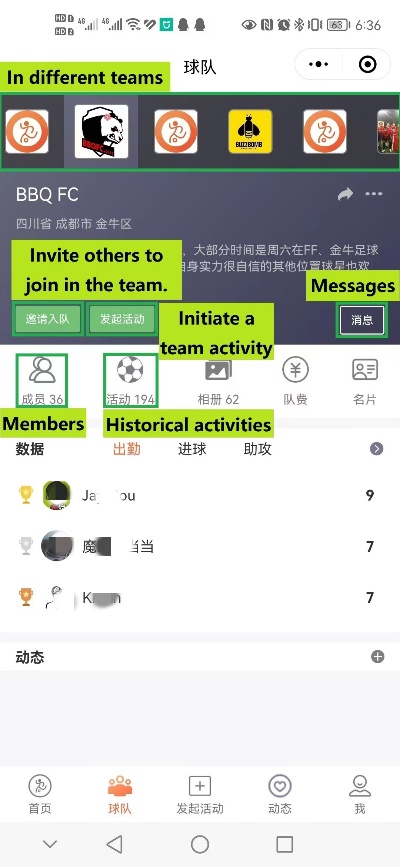


Figure C1. The homepage of the team BBQ FC in DaZhiYouQiu.


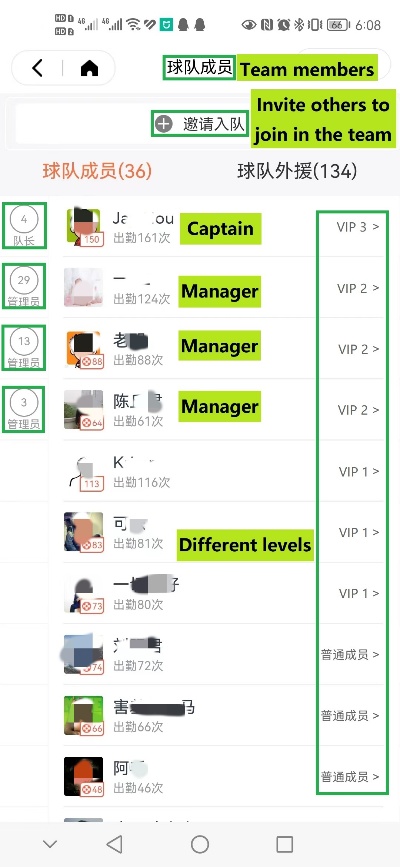


Figure C2. The member list of BBQ FC in DaZhiYouQiu.


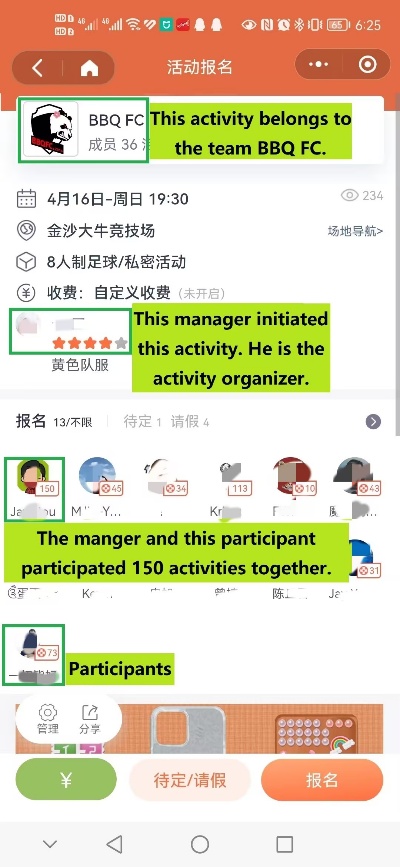


Figure C3. A team activity of BBQ FC in DaZhiYouQiu.

# Appendix D: Recognition, "Like", and Push Messages

Team identification is built on the "Like" function in the personal page. Team identification refers to the number of team members who mark "Like" on the team leader(s)' personal page. And we designed related functions to enrich the overall concept of team identification. For example, the organizer rating – the higher number of person-time participating in his/her activities, the higher the organizer rating, and a notification function related to "Like" – when the liked user publishes and signs up for public activity, his/her fans will receive a notification about it.

Figure D1 is a personal page of Pan in DaZhiYouQiu, from user Victor's perspective. Pan has the highest rating as he has organized activities for over 6k person-time, and Victor has marked him as "Like". Figure D2 shows that Victor is willing to receive the push messages to remind him of the public activities organized by Pan. The WeChat official account, with the same name of the applet, will send those messages, and Figure D3 gives an example.

In addition, we made a distinction in design between team identification and external interest in the focus team. External interest in the focus team can be expressed in the message board. Please refer to Appendix C, the first screenshots, where we marked "Message" is used for the expression for external interest.


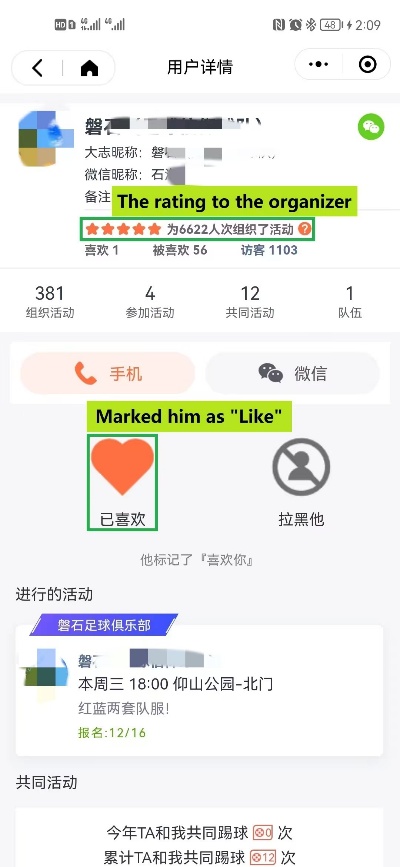


Figure D1. The personal page of Pan in DaZhiYouQiu, from user Victor's perspective.


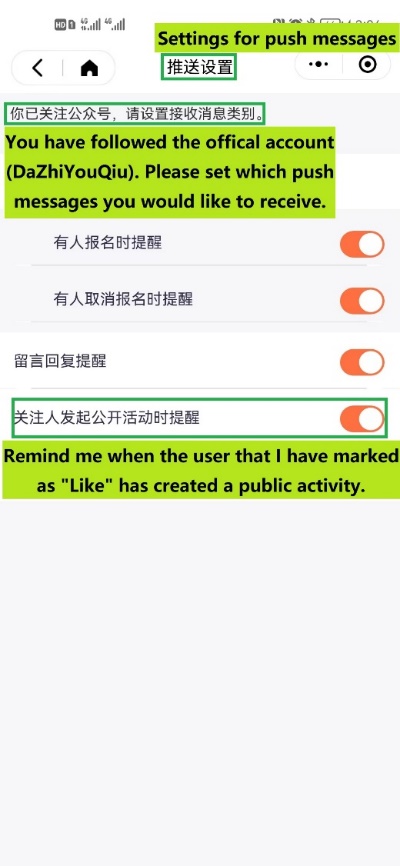


Figure D2. Victor's settings for push messages in DaZhiYouQiu.


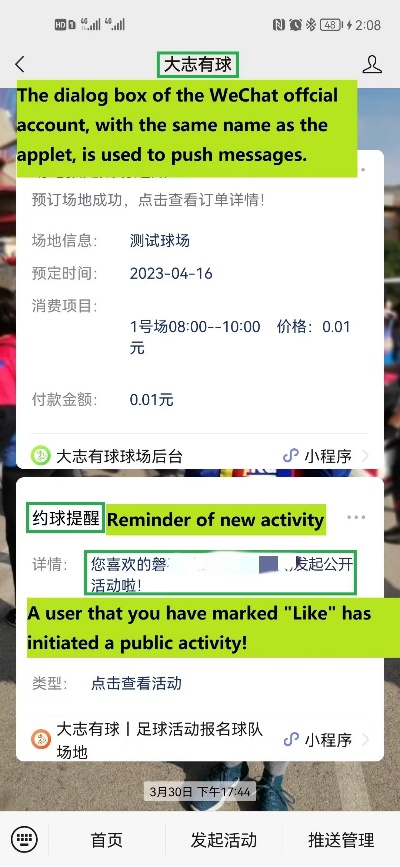


Figure D3. An example of push message about Pan's participation – from DaZhiYouQiu to Victor.

# Appendix E: Two Examples of Special Activities

Figure E1 shows an example of a cancelled activity because of insufficient participants. Figure E2 shows an opposite example, that is, an activity with twice as the usual participants. The activity announcement indicates that this activity was special when new jerseys and sponsors would be announced, which is why this activity had so many participants.


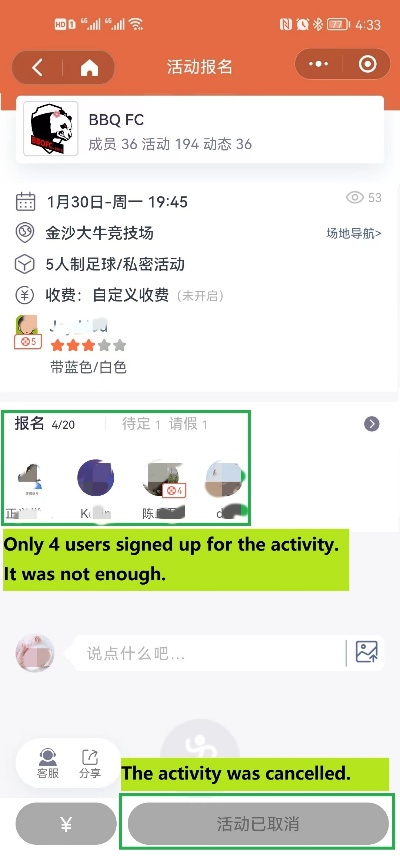


Figure E1. A cancelled activity in DaZhiYouQiu.


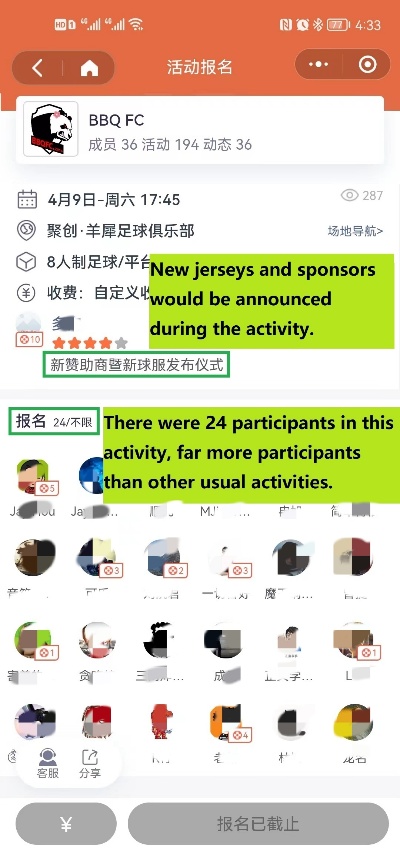


Figure E2. An activity with twice as the usual participants in BBQ FC.

# Appendix F: Robustness Testing

The robustness testing utilized data from May 2021, initially comprising over 5,000 platform activities. Applying identical filtering protocols established in Section 4.2 - exclusion of activities cancelled and activities with <5 participants, team-unaffiliated activities, and teams organizing <4 monthly activities - yielded a refined dataset of 241 teams conducting 1,385 valid activities, with 6,470 participants generating >7,000 team-member relations. Teams exhibited significant heterogeneity in activity patterns and geographic dispersion: during the one-month observation period, teams organized an average of 5.74 activities (SD = 6.42), with 17.66% classified as high-activity (>6 activities), 27.67% as medium-activity (5–6 activities), and 54.67% as low-activity (exactly 4 activities); geographically, samples spanned 21 cities across 15 Chinese provincial-level regions and Japan, dominated by high-cluster concentrations in Beijing (37.34%), Shanghai (11.12%), and Sichuan (10.09%), complemented by medium-distribution regions like Tianjin(8.81%) and Guandong(4.28%), while 17 teams (7.05%) had no location record.

Descriptive statistics are shown in Table F1, including means, standard deviations, and correlations of focal variables. Two-Sample Kolmogorov-Smirnov test indicates that this dataset is generally different from the previous one.

The results of this dataset are consistent with those of the previous dataset, as Table F3 and Figure F1 show stronger support for our findings. In contrast with our previous result, model 2 shows no significant effect of task-oriented interaction on team organizing efficiency (*β = .143, p = .530*), suggesting that the linear relationship is not stable. However, model 3 supports the inverted-U-shaped pattern between them (*β = –.384, p < .001*) with the inflection point at a task-oriented interaction value of 2.31, which corresponds to an actual task-oriented interaction value of 132.09. Thus, H1 is supported. In addition, model 4 shows the negative interaction of team identification with task-oriented interaction (*β = –1.478, p < .001*) and the positive interaction with the quadratic term (*β = .41, p < .001*), indicating the moderating role of team identification on task-oriented interaction’s effect. Therefore, H2 is supported. For better illustration, we plot Figure F1, which shows the considerable difference in task-oriented interaction’s impact on team organizing efficiency in terms of team identification.

Table F1. Descriptive statistics: means, standard deviations, and correlations of the variables.

| Variables | Mean | SD | 1 | 2 | 3 | 4 | 5 |
| --- | --- | --- | --- | --- | --- | --- | --- |
| 1. Team organizing efficiency | 14.195 | 3.613 |  |  |  |  |  |
| 2. Task-oriented interaction | 44.660 | 37.914 | .095^*^ |  |  |  |  |
| 3. Team identification | 1.531 | 3.037 | .231^***^ | .556^***^ |  |  |  |
| 4. Team duration | 389.241 | 240.349 | 0.260^***^ | .082 | .310^***^ |  |  |
| 5. New individual in team | .309 | .309 | –.130^*^ | –.002 | –.080 | –.515^***^ |  |
| 6. Multi-teamer in team | .094 | .152 | .221^***^ | .198^**^ | .324^***^ | .118^*^ | .131^*^ |

Note. 241 teams related to 6,470 individuals generated 7,201 individual-team relations with 1,385 activities, which implies that multiple team memberships existed. SD = standard deviation. ** p* < .05, *** p* < .01, **** p* < .001.

Table F2. Results of Two-Sample Kolmogorov-Smirnov test of our datasets.

| ***D_m, n, α_* = 0.128** | ***D_m, n_*** | **Verdict** |
| --- | --- | --- |
| Team organizing efficiency | 0.216 | Significantly different |
| Task-oriented interaction | 0.152 | Significantly different |
| Team identification | 0.548 | Significantly different |
| Team duration | 0.356 | Significantly different |
| New individual in team | 0.151 | Significantly different |
| Multi-teamer in team | 0.073 | No significant difference |
| Note. The null hypothesis is H0: both datasets come from a population with the same distribution. *D_m, n, α_* is the critical value that if *D_m, n_* > *D_m, n, α_*, we reject the null hypothesis (at significance level α). Here m = 214, the number of samples in the first dataset; n = 241, the number of samples in the current dataset for robustness testing; α = 0.05. For more details about Two-Sample Kolmogorov-Smirnov test, please see it in [www.real-statistics.com](https://real-statistics.com/non-parametric-tests/goodness-of-fit-tests/two-sample-kolmogorov-smirnov-test/) (accessed on April 26, 2023). | | |

Table F3. Regression results of team identification moderation on task-oriented interaction and team organizing efficiency.

| Variables | Model 1 | Model 2 | Model 3 | Model 4 |
| --- | --- | --- | --- | --- |
| Team duration | .765^**^ | .756^**^ | .565^*^ | .172 |
| New individual in team | –.172 | –.173 | –.254 | –.291 |
| Multi-teamer in team | .729^**^ | .702^**^ | .596^**^ | .232 |
| Task-oriented interaction |  | .143 | 1.771^***^ | 1.863^***^ |
| Team identification |  |  |  | 1.788^***^ |
| TOI^2^ |  |  | –.384^***^ | –.574^***^ |
| TOI×TI |  |  |  | –1.478^***^ |
| TOI^2^×TI |  |  |  | .211^***^ |
| *R^2^* | .106 | .107 | .218 | .345 |
| *ΔR^2^* | .106^***^ | .001 | .111^***^ | .127^***^ |

Note. Coefficients are unstandardized. Maximum VIF = 6.371, which is less than 10. DV, team organizing efficiency; TOI, task-oriented interaction; TI, team identification. ** p* < 0.05, *** p* < 0.01, **** p* < 0.001.

Figure F1. The curvilinear moderation of team identification on task-oriented interaction and team organizing efficiency.
